# Eustachian tube dysfunction: consensus statement on definition, types, clinical presentation and diagnosis

**DOI:** 10.1111/coa.12475

**Published:** 2015-09-07

**Authors:** AGM Schilder, MF Bhutta, CC Butler, C Holy, LH Levine, KJ Kvaerner, G Norman, RJ Pennings, D Poe, JT Silvola, H Sudhoff, VJ Lund

**Affiliations:** * evidENT, Ear Institute, University College LondonLondon, UK; † Royal National Throat Nose and Ear Hospital, University College London HospitalsLondon, UK; ‡ Nuffield Department of Primary Care Health Sciences, University of OxfordOxford, UK; § Acclarent IncMenlo Park, CA, USA; ¶ Cleveland ClinicCleveland, OH, USA; ** Department of Research and Education, Oslo University HospitalUllevål, Norway; †† BI Norwegian Business SchoolOslo, Norway; ‡‡ School of Nursing, Midwifery & Social Work, University of ManchesterManchester, UK; §§ Department of Otorhinolaryngology, Radboud University Nijmegen Medical CentreNijmegen, The Netherlands; ¶¶ Department of Otolaryngology, Boston Children's Hospital, Harvard Medical SchoolBoston, MA, USA; *** Department of Otorhinolaryngology, Oslo University HospitalOslo, Norway; ††† Department of Otorhinolaryngology, Head and Neck Surgery, Klinikum BielefeldBielefeld, Germany

## Introduction

A recent systematic review of treatments of Eustachian tube dysfunction commissioned by the UK NIHR Health Technology Assessment (HTA) Programme revealed that an important limitation with the available evidence is a lack of consensus on the definition and diagnosis of this disorder.^1^ The HTA report recommended that key to advancing research in this field is achieving consensus on diagnostic criteria for Eustachian tube dysfunction (to identify eligible patients for future trials) and on important clinical outcomes.

To address this need, an international forum of scientists and physicians with expertise in the field of Eustachian tube disorders met at a workshop in Amsterdam on 21 June 2014 and was tasked to come to an agreement on the definition, clinical presentation and diagnosis of Eustachian tube dysfunction, and areas for future research. This study summarises the outcomes of that meeting.

## Workshop design

A purposive sample of International experts in the field was brought together, spanning primary to tertiary care, and across the translational research pathway, from molecular to implementation science specialists.

The panel used the systematic review conducted for the UK NIHR HTA^1^ as the starting point. Consensus was achieved through a series of presentations by individual panel members and discussions around themes of function and dysfunction of the Eustachian tube, definitions, symptoms, signs and clinical investigation of Eustachian tube dysfunction. This study represents the consensus group opinion and was drafted and revised using an iterative process including all panel members.

The contribution of Eustachian tube dysfunction to mucosal or squamous forms of otitis media and the effectiveness of treatments for Eustachian tube dysfunction were outside the remit of this workshop. We did not consider disease in childhood, and so this statement refers only to disease in adults.

## Normal function of the Eustachian tube

The panel agreed that the Eustachian tube has unique functions and can be thought of as an organ; failure of its functions comprises dysfunction. The functions of the Eustachian tube are as follows^2^:

1. pressure equalisation and ventilation of the middle ear,
2. mucociliary clearance of secretions from the middle ear,
3. protection of the middle ear from sounds, and from pathogens and secretions from the nasopharynx.

Pressure in the middle ear is maintained through two mechanisms: middle ear mucosal gas exchange and opening of the Eustachian tube to equilibrate pressure with that in the nasopharynx.^3^ The relative contribution of these two mechanisms to normal middle ear ventilation is not known. Recent evidence suggests that, in the healthy middle ear, pressure slowly decreases, and periodic opening of the Eustachian tube restores the middle ear towards atmospheric pressure.^4^

Clearance of middle ear secretions occurs through both a muscular peristaltic action in the Eustachian tube and through the mucociliary escalator. When functioning normally, the Eustachian tube protects the middle ear against inflammation and infection by viruses, bacteria and gastro-oesophageal reflux.

## Definition of Eustachian tube dysfunction

The panel agreed that Eustachian tube dysfunction is a syndrome with a constellation of signs and symptoms suggestive of dysfunction of the Eustachian tube. This does not preclude that Eustachian tube dysfunction can also be a mechanism to middle ear disease.

Although in a strict sense Eustachian tube dysfunction is a failure to perform any of the Eustachian tube functions, in clinical practice, Eustachian tube dysfunction usually refers to a problem with the ventilatory function of the Eustachian tube. As such, Eustachian tube dysfunction is defined by symptoms and signs of pressure dysregulation in the middle ear.

The panel agreed to distinguish acute Eustachian tube dysfunction, transient with symptoms and signs for less than 3 months, from chronic dysfunction, symptoms and signs for more than 3 months. We agreed that there are three subtypes of Eustachian tube dysfunction:

1. dilatory Eustachian tube dysfunction,
2. baro-challenge-induced Eustachian tube dysfunction,
3. patulous Eustachian tube dysfunction.

Dilatory Eustachian tube dysfunction can be broken down as follows:

1. functional obstruction,
2. dynamic dysfunction (muscular failure),
3. anatomical obstruction.

Current ICD-10 codes for Eustachian tube dysfunction include the following: H68.0 inflammatory dilatory dysfunction of the Eustachian tube, H68.1 obstruction of the Eustachian tube, H69.0 patulous Eustachian tube, H69.8 other defined Eustachian tube dysfunction and H69.9 non-defined Eustachian tube dysfunction. We propose that future coding should consider the new classification system suggested here.

## Clinical history: symptoms of Eustachian tube dysfunction

To diagnose Eustachian tube dysfunction, the patient must present with symptoms of pressure disequilibrium in the affected ear, specifically symptoms of ‘aural fullness’ or ‘popping’ or discomfort/pain. Patients may also report pressure, clogged or ‘under water’ sensation, crackling, ringing, autophony and muffled hearing.

Acute dilatory Eustachian tube dysfunction is often preceded by an upper respiratory tract infection, or sometimes by an exacerbation of allergic rhinitis, which presumably causes inflammation in the Eustachian tube orifice or lumen. Some patients may have a prior history of otitis media. It is not clear whether the aetiology of chronic dilatory Eustachian tube dysfunction is an extension of the same pathology underlying acute dilatory Eustachian tube dysfunction, or whether other pathological mechanisms may underlie these symptoms. Some patients with dilatory Eustachian tube dysfunction may report repeated Valsalva or jaw-thrust manoeuvres in an attempt to equalise negative middle ear pressure; others describe altered hearing or tinnitus.

In baro-challenge-induced Eustachian tube dysfunction, symptoms of aural fullness, popping or discomfort/pain occur, or are initiated, under conditions of alteration to the ambient pressure. Typically symptoms may manifest when scuba-diving or on descent from altitude, but can also occur under conditions of less marked ambient pressure fluctuation. Patients are typically asymptomatic once they return to ground level, although significant baro-challenge may cause temporary middle ear effusion or haemotympanum.

Patulous Eustachian tube dysfunction presents with symptoms of aural fullness and autophony. Symptoms may be better in the supine position or during upper respiratory tract infection.^4^ They may worsen during exercise. Patulous Eustachian tube dysfunction is thought to be caused by an abnormally patent Eustachian tube; as such, it may be precipitated by recent weight loss, although in the majority of cases no underlying precipitating event is evident. Some patients with patulous Eustachian tube dysfunction will habitually sniff.

## Clinical assessment: signs of Eustachian tube dysfunction

Clinical assessment will vary depending upon what investigations are readily available (e.g. in primary care, tympanometry is rarely available). Ideally assessment should include the following:

1. otoscopy or otomicroscopy,
2. tympanometry,
3. Rinne's and Weber's tuning fork tests or pure tone audiometry,
4. nasopharyngoscopy (to visualise the opening of the Eustachian tube).

It was agreed that to diagnose dilatory Eustachian tube dysfunction, patient-reported symptoms should go together with evidence of negative pressure in the middle ear as assessed by clinical assessment, either as follows:

1. otoscopic or otomicroscopic evidence of tympanic membrane retraction and/or
2. tympanogram indicating negative middle ear pressure.

An ability to auto-inflate the middle ear on Valsalva or Toynbee manoeuvre confirms some degree of patency of the Eustachian tube, but the panel felt that ability to auto-inflate is not sufficiently sensitive or specific for Eustachian tube dysfunction to have clinical utility.

In baro-challenge-induced Eustachian tube dysfunction, otoscopy and tympanometry may be normal at normal ambient pressure, and so diagnosis relies on patient history. In some cases of baro-challenge-induced Eustachian tube dysfunction, middle ear effusion or haemotympanum may be evident.

In Patulous Eustachian tube dysfunction, symptoms go together with evidence on otoscopy or tympanometry of tympanic membrane excursion with breathing.^4^

Tympanometry may not be available in a primary care setting, in which case diagnosis of Eustachian tube dysfunction is confirmed by abnormal otoscopy or may be presumptive. If symptoms of Eustachian tube dysfunction are chronic (more than 3 months) and/or troublesome, referral to secondary care should be considered to confirm the diagnosis and to determine its cause.

Pure tone audiometry should include air and bone conduction thresholds. A mild or moderate conductive hearing loss may be found in some patients with Eustachian tube dysfunction. In primary care, tuning fork tests (Rinne's and Weber's tests) may be used as a substitute for audiometry, although these tests are less reliable.

Nasopharyngoscopy is usually only available in secondary care. Examination may reveal a cause for Eustachian tube dysfunction, for example inflammation adjacent to the Eustachian tube orifice, or (rarely) neoplasms, scarring or other lesions.

The panel agreed that radiological evaluation does not routinely play a role in diagnosis of Eustachian tube dysfunction, and should be reserved for cases where additional or alternate pathology is suspected based upon history or examination.

**Fig. 1:**
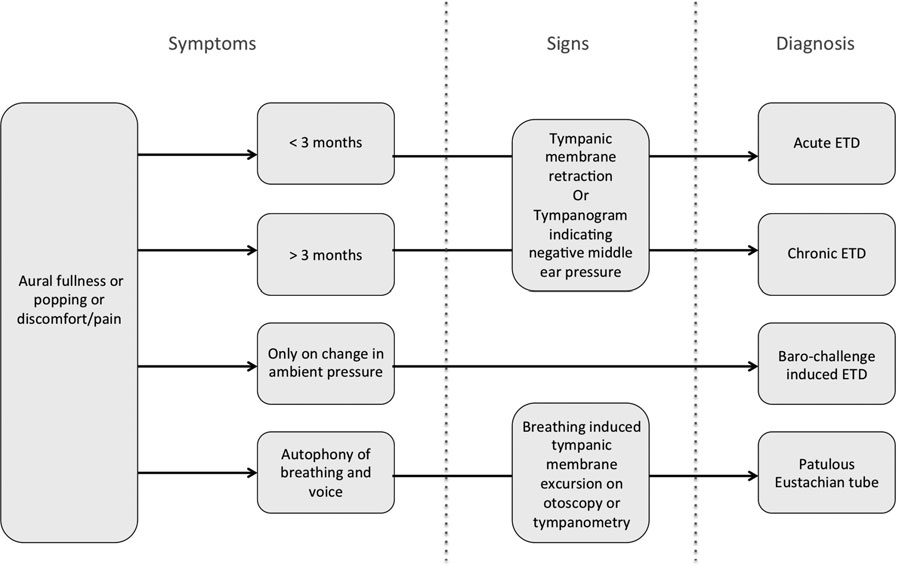
Symptoms and signs used to define Eustachian tube dysfunction (ETD) and the subtypes of acute ETD, chronic ETD, baro-challenge-induced ETD and patulous Eustachian tube.

The combination of clinical symptoms and signs enables a diagnostic algorithm for the diagnosis and subclassification of Eustachian tube dysfunction (Fig.1).

## Role of Eustachian tube function tests and scoring systems

The panel agreed that at the current time, there is no universally accepted set of patient-reported symptom scores, functional tests or scoring systems to diagnose Eustachian tube dysfunction, and the diagnosis should therefore at this stage rely on the clinical observations (symptoms and signs) detailed above.

A number of tests of the ventilatory function of the Eustachian tube have been devised, including tubomanometry, sonotubometry, nine-step inflation–deflation test and pressure chamber tests. At the current time, the equipment these tests require is not widely available, and their accuracy and validity is unclear,^5^ but they can be useful research tools.

The Eustachian Tube Dysfunction Questionnaire (ETDQ-7)^6^ scores symptoms of Eustachian tube dysfunction and is the only patient-reported outcomes tool to have undergone initial validation studies. The Eustachian Tube Score (ETS) and its extension the ETS-7^7^ combine subjective (clicking sound when swallowing, Valsalva) and objective (tubomanometry, tympanometry) measures of Eustachian tube function.

The panel agreed that there is a need for wider experience in the use of these instruments across centres, and for validation of these instruments using the criteria for diagnosis recommended in this study.

## Outcome measures

The panel agreed that in any future clinical trials, clinical outcomes should be assessed at baseline and in the short term (defined as 6 weeks to 3 months) and the long-term term (defined as 6–12 months), and should include assessment of patient-reported symptoms, otoscopy, tympanometry and pure tone audiometry.

## Differential diagnosis

Eustachian tube dysfunction should not be used to describe disease more properly classified as otitis media, including chronic otitis media with effusion (glue ear), chronic suppurative otitis media, tympanic membrane retraction and cholesteatoma. Whereas ventilatory dysfunction of the Eustachian tube may contribute to the onset or persistence of these types of otitis media, the relative importance of this contribution is a matter of debate and a debate outside of the remit of this work.

A number of other disorders can present with symptoms similar to Eustachian tube dysfunction. Patients with cochlear hydrops may describe periodic unilateral pressure sensation associated with altered hearing that typically lasts a few hours. Patients with temporomandibular joint (TMJ) dysfunction describe discomfort in front of and around the ear, typically unilateral, and in some cases associated with clicking or popping noises and altered hearing or tinnitus. Although there are no clear diagnostic criteria for TMJ dysfunction, aggravation of pain by manipulation or function of the jaw is a cardinal sign. In diagnosing patulous Eustachian tube dysfunction, other causes of autophony should be considered, including a fistula of the inner ear, for example due to superior semicircular canal dehiscence. Tullio phenomenon may suggest an inner ear fistula, although in isolation this sign is not reliable for diagnosis.

## Recommendations for future research

The definitions, diagnostic criteria and subclassification of Eustachian tube dysfunction presented in this consensus statement can be used to inform future research in this field. In particular, consensus and consistency in disease definition should enable better studies of the epidemiology of Eustachian tube dysfunction, and clear inclusion criteria and outcome measures for new clinical trials of treatments for Eustachian tube dysfunction.

Areas for future research include the following:

1. The epidemiology of Eustachian tube dysfunction, including prevalence of associated symptoms in the community, primary care, and hospital populations, natural history, psychosocial impact and relation to preceding or subsequent otitis media.
2. Further work to develop and validate patient-reported symptom scores and subjective and objective pressure tests, as instruments to aid diagnosis and to assess disease severity and treatment outcomes.
3. Working with patients and the public to develop a core set of outcome measures to monitor the effects of treatments of Eustachian tube dysfunction in a research and clinical setting.
4. Randomised controlled trials of treatments for Eustachian tube dysfunction, incorporating recommendations regarding the definitions, subtypes, diagnostic criteria and outcome measures of Eustachian tube dysfunction presented here.

**Keypoints**

- A recent systematic review showed that there is wide variation in diagnostic criteria for Eustachian tube dysfunction.
- An expert panel was convened to define this disorder in adults, and agreed that there are probably three subtypes of Eustachian tube dysfunction: dilatory, baro-challenge induced, and patulous.
- Eustachian tube dysfunction presents with symptoms of pressure disequilibrium in the affected ear(s).
- In dilatory dysfunction there are signs on otoscopy or tympanometry of negative middle ear pressure. In baro-challenge induced dysfunction, symptoms occur only on changes to ambient pressure. In patulous dysfunction there is otoscopic or tympanometric evidence of excursion of the tympanic membrane with breathing.
- The diagnostic categories and criteria detailed in this paper may be used in future studies of epidemiology, psychosocial impact, and treatment.
